# Technophobia and Its Associated Factors Among Registered Nurses in China: A Cross‐Sectional Multicenter Study

**DOI:** 10.1155/jonm/9935996

**Published:** 2026-04-16

**Authors:** Xuange Sun, Xu Liu, Xiaolin Chang, Na Hu, Xiangxiu Qi, Xiaofei Li

**Affiliations:** ^1^ Department of Endocrinology and Metabolism, The First Hospital of China Medical University, Shenyang, China, cmu.edu.cn; ^2^ School of Nursing, China Medical University, Shenyang, China, cmu.edu.tw; ^3^ Transplantation and Hepatobiliary Department, The First Hospital of China Medical University, Shenyang, China, cmu.edu.cn; ^4^ Outpatient Service by Famous Specialists, The First Hospital of China Medical University, Shenyang, China, cmu.edu.cn; ^5^ Hospital Infection Control Office, Shengjing Hospital of China Medical University, Shenyang, China, cmu.edu.cn

**Keywords:** digital health, job satisfaction, nurses, psychosocial factors, sleep quality, social support, technophobia, workload

## Abstract

**Aim:**

To examine the level of technophobia and identify its associated sociodemographic, occupational, and psychosocial factors among registered nurses in China.

**Background:**

The rapid digital transformation of healthcare systems demands that nurses effectively adopt and use digital technologies. Technophobia—defined as negative emotional reactions toward technology—may impede nurses’ adaptation to technological innovations. However, no study is currently available on technophobia and its associated factors in registered nurses.

**Design:**

A multicenter, cross‐sectional study.

**Methods:**

A total of 1559 registered nurses from two university‐affiliated tertiary hospitals in Northeast China were surveyed between September and October 2025. Data were collected using a self‐administered questionnaire including sociodemographic factors, occupational factors, the validated Chinese version of the Technophobia Scale, the Multidimensional Scale of Perceived Social Support, and the Athens Insomnia Scale. A generalized linear model was fitted to identify independent predictors of technophobia.

**Results:**

The nurses’ median technophobia score was 26.00 (P_25_–P_75_: 13.00–39.00), indicating a low‐to‐moderate level overall. The generalized linear model revealed that male gender (*β* = 0.201), having one child (*β* = 0.130), living alone (*β* = 0.091), having a higher perceived workload (*β* = 0.048), and poorer sleep quality (*β* = 0.015) were independently associated with greater technophobia. Conversely, higher job satisfaction (*β* = −0.081) and stronger social support (*β* = −0.003) were independently associated with lower technophobia; however, the effect size for social support was relatively small, all *p* < 0.05.

**Conclusions:**

Technophobia among registered nurses in China is at a moderately low level. Interventions targeting modifiable factors—such as workload management, sleep health, job satisfaction, and social support—through structured digital literacy training, peer support mechanisms, and other supportive workplace strategies may help mitigate technophobia and enhance nurses’ readiness for digital transformation.

**Patient or Public Contribution:**

Nurses voluntarily participated in the survey. The findings may indirectly benefit patients through improved nurse engagement with digital health systems and safer, more efficient care delivery.

## 1. Introduction

The rapid advancement of digital technologies has profoundly transformed healthcare delivery worldwide. Electronic health records, telemedicine, artificial intelligence‐assisted diagnostics, and mobile health applications have become integral components of modern clinical practice, contributing to improved efficiency, accuracy, and patient safety [1–3]. In China, the national push for digital health and information systems has accelerated the digital transformation of healthcare institutions [4]. Recent reports indicate that over 90% of tertiary hospitals have completed deployment of basic information management systems, reflecting the government’s strong policy support for health informatization [5]. In nursing, technology integration has facilitated evidence‐based decision‐making, enhanced monitoring and documentation, and expanded patient education and communication channels [6–8]. Nurses in China are increasingly expected to acquire digital competencies and adapt to rapidly changing technological environments.

In recent years, nurses have played a crucial role in guiding the integration of digital technologies into healthcare settings [9]. However, the mandatory and widespread adoption of digital technologies has significantly increased the pressure on nurses to learn and effectively use these systems [10, 11]. The digital shift has made it increasingly difficult for nurses to balance traditional care duties with the demands of mastering new technology, creating a unique form of job‐related anxiety: technophobia [12]. Technophobia—defined as aversion or anxiety toward technologies and technology‐related products—represents a form of technology‐related anxiety [13]. In healthcare settings, technophobic tendencies among nurses may result in reduced engagement in technology‐based interventions, hinder the effective utilization of information systems, and lead to the avoidance of innovative digital tools [14], thereby reducing job satisfaction, compromising the quality of patient care, and impeding the advancement of nursing practice.

Previous research has shown that technophobia is associated with a range of demographic and psychosocial factors. Age, living situation, health status, education level, monthly income, and e‐health literacy have all been identified as factors related to technophobia [15, 16]. In addition, psychosocial factors may also play an important role in shaping individuals’ attitudes toward technology. Among these factors, social support has been identified as an important resource that facilitates adaptation to technological change by providing emotional encouragement and practical assistance [17]. Conversely, sleep problems such as insomnia may impair cognitive functioning, emotional regulation, and learning ability, which are critical for adapting to new technologies in healthcare environments [18]. However, evidence regarding technophobia has mainly focused on older adults [15, 17], university students [19, 20], and teachers [16, 21]. Nurses—who constitute nearly half of the global health workforce [22], serve as the frontline of patient care, and are pivotal for the successful integration of digital technologies into clinical practice [9]—have been largely overlooked in research on technophobia. Addressing this gap is critical, as nurses’ readiness and confidence with digital tools directly impact patient safety, care quality, and the sustainable adoption of healthcare innovations. Therefore, this study aims to investigate the current status of technophobia and its associated factors among nurses in China using a cross‐sectional multicenter design. By identifying the demographic, occupational, and psychosocial determinants of technophobia, this study seeks to provide evidence to guide targeted interventions that enhance nurses’ digital readiness, reduce technology‐related anxiety, and thereby promote the sustainable integration of digital health innovations into nursing practice.

## 2. Methods

### 2.1. Study Setting and Participants

This study employed a cross‐sectional design and collected questionnaires using the electronic “Questionnaire Star” tool (https://www.wjx.cn/). The participants were recruited from two tertiary hospitals in China’s Northeast, namely the First Hospital of China Medical University and the Shengjing Hospital of China Medical University. All the nurses of these two hospitals who met the inclusion criteria and did not meet the exclusion criteria were invited to participate in this survey via WeChat between September 2025 and October. The inclusion criteria were (1) registered nurses employed at the respective hospital; (2) engaged in clinical or related nursing work for more than 6 months and possessing basic nursing experience; (3) voluntarily agreed to participate in the study; and (4) able to independently complete the electronic questionnaire. Nurses currently on extended leave (e.g., maternity leave, sick leave) and not in regular work were excluded.

This study has been carried out in strict adherence to the Declaration of Helsinki and has passed the ethical approval of the Ethics Committee of the First Hospital of China Medical University, number [2025] 782. Informed consent from all participants was obtained before they participated in this study.

### 2.2. Sample Size Determination

An a priori sample size estimation was conducted using G∗Power 3.1 for multiple linear regression (F tests, linear multiple regression: fixed model, *R* ^2^ deviation from zero). A small‐to‐moderate effect size (*f* ^2^ = 0.02), *α* = 0.05, power = 0.95, and 16 predictors were assumed. The calculation indicated a minimum required sample size of 204 participants. Considering a possible 20% attrition rate, the final required sample size was 255. A total of 1561 nurses initially participated in the survey. After data screening, two questionnaires were excluded due to abnormal values in the age variable. Therefore, data from 1559 participants were included in the final analysis.

### 2.3. Measures

#### 2.3.1. Questionnaire for General Information

The general information of this study includes age (years), gender, marriage, education level, family per capita monthly income, number of children, living status, department, employment status, professional title, number of night shifts per week, perceived staffing adequacy status, perceived workload, and job satisfaction.

#### 2.3.2. Technophobia

The technophobia was measured using the validated Chinese version of the Technophobia Scale [23]. The original scale was compiled by Khasawneh [13] and included 16 items and five dimensions named techno paranoia, techno fear, techno anxiety, cybernetic revolt, and communication devices avoidance [13]. The original scale demonstrated good internal consistency, with a Cronbach’s *α* coefficient of 0.867. During the localization process, item 5 and item 8 were excluded due to low relevance (*r* < 0.4), and item 13 was excluded due to multiple meanings [23]. The final Chinese‐language version of the scale comprises 13 items across three dimensions, including techno‐anxiety, techno‐paranoia, and privacy concerns. The Chinese version demonstrated good psychometric properties, with a Cronbach’s *α* of 0.911 for the total scale and 0.759–0.885 for the subscales. Each item was rated on a 5‐point Likert scale ranging from 1 (strongly disagree) to 5 (strongly agree), yielding a total score between 13 and 65. Higher scores indicate greater levels of technophobia. The Technophobia Scale was initially validated among older adults and has subsequently been applied to university students [19, 20] and teachers [21]. Its items capture general negative emotional reactions to technology, making the scale suitable for adult populations. Representative items include: “I feel anxious when I have to use new communication devices (e.g., a new mobile phone or a newly purchased computer),” “I try to avoid using new technologies, such as smartphones and smart wristbands, as much as possible,” and “I am very afraid that technology will change our way of life, communication patterns, and emotional relationships.” In the present study, the Chinese version demonstrated good internal consistency (Cronbach’s *α* = 0.984), supporting its reliability in the nurse sample.

#### 2.3.3. Social Support

Social support was measured using the Multidimensional Scale of Perceived Social Support, which includes 12 items divided into family support, friend support, and other support, three dimensions [24]. The original scale demonstrated good internal consistency, with Cronbach’s *α* coefficients ranging from 0.85 to 0.91 across subscales and 0.88 for the total scale. The Chinese version of the scale, translated by Jiang [25], has been widely used in Chinese nurse populations and has demonstrated good reliability and validity in previous studies [26, 27]. Each item was rated on a 7‐point Likert scale ranging from 1 (strongly disagree) to 7 (strongly agree), resulting in a total score ranging from 12 to 84. Higher scores indicate greater social support. In this study, it demonstrated high internal consistency, with a Cronbach’s alpha of 0.984.

#### 2.3.4. Insomnia Status

The sleep quality was measured using the Athens Insomnia Scale (AIS) [28]. The original scale demonstrated good reliability with Cronbach’s *α* = 0.89. The Chinese version of the scale has been widely used in Chinese populations and has demonstrated good reliability and validity in previous research [29, 30]. It covers eight items and two dimensions, including nocturnal sleep difficulties and daytime consequences of disturbed sleep. Each item is rated on a 0‐3 scale, with 0 indicating no impact and 3 indicating significant impact, yielding a total score ranging from 0 to 24, with higher scores indicating more severe insomnia symptoms, with a Cronbach’s alpha of 0.903.

### 2.4. Statistical Analysis

Data were analyzed using SPSS version 25.0. A two‐sided *p* value < 0.05 was considered statistically significant. Normality of the sample distribution was tested using qq‐plots and the Kolmogorov–Smirnov test. Median and quartile spacing were used to describe the continuous variables in this study because the variables were not normally distributed, and the percentages (constituent ratios) were used to describe the qualitative data. For univariate comparisons, the Mann–Whitney *U* test or Kruskal–Wallis test was used. Spearman correlation analysis was used to test for the relationship between technophobia and the continuous variables. To identify factors associated with technophobia, a generalized linear model (GLM) with a gamma distribution and log link function was fitted. The assessment of multicollinearity potential was conducted using tolerance and the variance inflation factor (VIF). Noncollinearity was accepted if the tolerance exceeded 0.10 and the VIF was below 10.0.

## 3. Results

### 3.1. Characteristics of the Study Sample

In total, 1559 participants were included in this study. The median technophobia score of Chinese nurses was 26.00 (P_25_: 13.00, P_75_: 39.00). The median age of participants was 37.0 (P_25_: 30.00, P_75_: 40.00). The median perceived workload scores were 3.00 (P_25_:2.00, P_75_: 3.00), and the median job satisfaction scores were 4.00 (P_25_: 4.00, P_75_: 4.00). The descriptive statistics of variables are shown in Table 1.

**TABLE 1:** Characteristics of the study sample.

Variable	*M* (P_25_, P_75_)/frequency (%)
Technophobia	26.00 (13.00, 39.00)
Age	37.00 (30.00, 40.00)
Perceived workload	3.00 (2.00, 3.00)
Job satisfaction	4.00 (4.00, 4.00)
Sleep quality	6.00 (2.00, 9.00)
Social support	72.00 (60.00, 82.00)
Gender	
Male	47 (3.01)
Female	1512 (96.99)
Marriage	
have a spouse	1079 (69.21)
have no spouse	480 (30.79)
Education level	
college and below	90 (5.77)
undergraduate	1435 (92.05)
Master’s and above	34 (2.18)
Family per capita monthly income (yuan)	
< 8000	202 (12.96)
8000–10000	485 (31.11)
> 10,000	872 (55.93)
Number of children	
0	531 (34.06)
1	804 (51.57)
> 1	224 (14.37)
Living status	
living with others	1257 (80.63)
living alone	302 (19.37)
Department	
outpatient	107 (6.86)
ICU	81 (5.20)
medical ward	274 (17.58)
surgical ward	319 (20.46)
operating room	39 (2.50)
emergency	52 (3.34)
pediatrics/neonatology	320 (20.53)
others	367 (23.54)
Employment status	
permanent staff with official establishment *(bianzhi)*	113 (7.25)
contract staff	1439 (92.30)
agency hired/dispatched staff	7 (0.45)
Professional title	
staff nurse	1055 (67.67)
supervisor nurse	494 (31.69)
associate senior nurse/chief nurse	10 (0.64)
Number of night shifts per week	
0	483 (30.98)
1	457 (29.31)
2	521 (33.42)
3	98 (6.29)
Perceived staffing adequacy	
adequate	827 (53.05)
moderate	625 (40.09)
inadequate	107 (6.86)

### 3.2. Univariate Analysis of Technophobia in Nurses

There were significant differences in the technophobia in terms of the number of children and perceived staffing adequacy (*p* <  0.05) (Table 2).

**TABLE 2:** Comparison of technophobia scores among nurses with different sociodemographic and work‐related characteristics.

Variable	*M* (P_25_, P_75_)	*Z/H*	*p*
Gender			
Male	27.00 (15.50, 43.50)	*Z* = −1.327	0.185
Female	26.00 (13.00, 39.00)		
Marriage			
have a spouse	26.00 (13.00, 39.00)	*Z* = −1.313	0.189
have no spouse	26.00 (13.75, 37.00)		
Education level			
college and below	26.00 (14.25, 39.00)	*H* = 1.549	0.461
undergraduate	26.00 (13.00, 39.00)		
Master’s and above	22.00 (13.00, 34.75)		
Family per capita monthly income (yuan)			
< 8000	26.00 (15.00, 39.00)	*H* = 2.408	0.300
8000–10000	26.00 (14.00, 39.00)		
> 10,000	26.00 (13.00, 39.00)		
Number of children			
0	26.00 (13.00, 35.00)	*H* = 10.191	0.006
1	26.00 (14.00, 39.00)		
> 1	26.00 (13.00, 38.00)		
Living status			
living with others	26.00 (13.00, 39.00)	*Z* = −0.358	0.720
living alone	26.00 (14.00, 39.00)		
Department			
outpatient	26.00 (14.00, 39.00)	*H* = 3.511	0.834
ICU	26.00 (13.00, 39.00)		
medical ward	26.00 (13.00, 34.00)		
surgical ward	26.00 (13.00, 37.00)		
operating room	24.00 (14.00, 37.00)		
emergency	26.00 (17.00, 35.00)		
pediatrics/neonatology	26.00 (15.00, 39.00)		
others	26.00 (13.00, 39.00)		
Employment status			
permanent staff with official establishment *(bianzhi)*	30.00 (17.00, 39.00)	*H* = 5.288	0.071
contract staff	26.00 (13.00, 39.00)		
agency hired/dispatched staff	19.00 (13.00, 30.50)		
Professional title			
staff nurse	26.00 (13.00, 39.00)	*H* = 0.361	0.835
supervisor nurse	26.00 (13.00, 39.00)		
associate senior nurse/chief nurse	26.50 (14.75, 37.00)		
Number of night shifts per week			
0	26.00 (13.00, 39.00)	*H* = 4.455	0.216
1	26.00 (13.00, 38.00)		
2	26.00 (13.00, 39.00)		
3	27.00 (18.00, 39.00)		
Perceived staffing adequacy			
adequate	24.00 (13.00, 35.00)	*H* = 31.194	< 0.001
moderate	27.00 (17.00, 39.00)		
inadequate	26.00 (13.00, 41.50)		

Spearman correlation analysis was conducted to examine the associations between continuous variables and technophobia among nurses (Table 3). The results showed that age (*r* = 0.058, *p* = 0.021) was weakly but significantly positively correlated with technophobia. Perceived workload (*r* = 0.194, *p* < 0.001) and sleep quality (*r* = 0.252, *p* < 0.001) were also positively correlated with technophobia. Job satisfaction (*r* = −0.263, *p* < 0.001) and social support (*r* = −0.300, *p* < 0.001) were negatively correlated with technophobia.

**TABLE 3:** Correlation analysis between continuous variables and technophobia in nurses.

Variable	Technophobia	
	*r*	*p*
Age	0.058	0.021
Perceived workload	0.194	< 0.001
Job satisfaction	−0.263	< 0.001
Sleep quality	0.252	< 0.001
Social support	−0.300	< 0.001

### 3.3. Multivariate Analysis of Technophobia in Nurses

A GLM was performed to identify factors associated with nurses’ technophobia (Table 4). The results indicated that male nurses reported a higher technophobia level than female nurses (*β* = 0.201, 95% Cl: 0.054, 0.348, *p* = 0.007), although the magnitude of this association was modest. Nurses with one child had significantly severe technophobia compared with those without children (*β* = 0.130, 95% Cl: 0.037, 0.223, *p* = 0.006). In addition, living alone was positively associated with technophobia (*β* = 0.091, 95% Cl: 0.004, 0.179, *p* = 0.040), although the effect size was small. Higher perceived workload was positively associated with technophobia (*β* = 0.048, 95% Cl: 0.015, 0.081, *p* = 0.005), whereas higher job satisfaction was negatively associated with technophobia (*β* = −0.081, 95% Cl: −0.112, −0.050, *p* < 0.001). Sleep quality problems were also a significant associated factor of technophobia (*β* = 0.015, 95% Cl: 0.009, 0.020, *p* < 0.001). In addition, greater social support was associated with lower levels of technophobia (*β* = −0.003, 95% Cl: −0.005, −0.001, *p* = 0.008), although the magnitude of this association was very small, suggesting limited practical significance.

**TABLE 4:** Multiple linear regression analysis of technophobia.

Variables	*β* estimate with 95% CI	*p*	Tolerance	VIF
Gender (Ref: Female)	0.201 (0.054,0.348)	0.007	0.901	1.110
Marriage (Ref: have no spouse)	−0.005 (−0.099, 0.088)	0.912	0.301	3.318
Education level (Ref: college and below)				
undergraduate	0.033 (−0.079, 0.145)	0.562	0.622	1.608
postgraduate and above	−0.098 (−0.300, 0.104)	0.342	0.653	1.532
Number of children (Ref: 0)				
1	0.130 (0.037, 0.223)	0.006	0.267	3.751
> 1	0.093 (−0.017, 0.204)	0.096	0.385	2.595
Family per capita monthly income (yuan) (Ref: < 5000)				
5000–8000	−0.015 (−0.096, 0.067)	0.721	0.399	2.506
> 8000	−0.020 (−0.100, 0.059)	0.613	0.368	2.717
Living status (Ref: living with others)	0.091 (0.004, 0.179)	0.040	0.489	2.046
Department (Ref: outpatient)				
ICU	−0.111 (−0.255, 0.034)	0.134	0.551	1.814
medical ward	−0.014 (−0.127, 0.098)	0.802	0.311	3.219
surgical ward	0.007 (−0.106, 0.119)	0.909	0.275	3.641
operating room	−0.010 (−0.188, 0.169)	0.914	0.736	1.359
emergency	−0.132 (−0.299, 0.036)	0.123	0.625	1.600
pediatrics/neonatology	−0.029 (−0.138, 0.079)	0.594	0.297	3.369
others	0.057 (−0.049, 0.163)	0.294	0.281	3.558
Employment status (Ref: permanent staff with official establishment (bianzhi))				
contract staff	−0.096 (−0.208, 0.017)	0.096	0.619	1.615
agency hired/dispatched staff	−0.256 (−0.625, 0.112)	0.173	0.929	1.076
Professional title (Ref: staff nurse)				
supervisor nurse	−0.034 (−0.099, 0.030)	0.299	0.630	1.588
associate senior nurse/chief nurse	−0.038 (−0.354,0.278)	0.814	0.876	1.141
Number of night shifts per week (Ref: 0)				
1	−0.011 (−0.077, 0.055)	0.741	0.625	1.600
2	−0.021 (−0.090, 0.048)	0.546	0.536	1.864
3	0.077 (−0.032, 0.185)	0.167	0.804	1.244
Perceived staffing adequacy (Ref: adequate)				
moderate	0.044 (−0.009, 0.097)	0.102	0.837	1.195
inadequate	0.009 (−0.093, 0.112)	0.857	0.841	1.188
Age	0.000 (−0.006, 0.007)	0.903	0.282	3.544
Perceived workload	0.048 (0.015, 0.081)	0.005	0.774	1.293
Job satisfaction	−0.081 (−0.112, −0.050)	< 0.001	0.767	1.303
Sleep quality	0.015 (0.009, 0.020)	< 0.001	0.790	1.267
Social support	−0.003 (−0.005, −0.001)	0.008	0.786	1.272

*Note: β*, standardized regression coefficient.

Abbreviations: CI, confidence interval; VIF, variance inflation factor.

## 4. Discussions

This study examined the level of technophobia among nurses in China and explored its associated factors. The findings showed that technophobia among nurses was at a lower‐middle level, suggesting that there is still room for improvement in nurses’ adaptation to digital technologies. Several demographic, occupational, and psychosocial factors were associated with technophobia. These findings provide insight into potential factors influencing nurses’ adaptation to rapidly evolving healthcare technologies.

The results of this study showed that the median technophobia score of Chinese nurses was 26.00, which was at a lower‐middle level compared with the total score of 65.00 on the scale. This finding differed from the results of the study examining older Chinese individuals [15] and older Chinese patients with ischemic stroke [17], where technophobia scores were at the upper‐middle level. The possible reasons for the variation may be partly due to differences in age groups and different education levels. In our study, the median age of the nurses was 37.00 years. Younger individuals are often referred to as “digital natives”; they generally tend to be more comfortable with technology and may have had more exposure to digital technology throughout their education and early career [31]. In addition, a total of 94.22% of nurses had a bachelor’s degree or higher in this study, which suggests that they may have had more opportunities to engage in technology‐related training and knowledge acquisition during their academic years. Higher educational levels are also typically associated with greater cognitive resources, enabling individuals to process and adapt to new information more effectively [32, 33]. Still, technophobia remains an important issue within the nursing population, particularly when confronted with complex medical technologies and information systems.

The study revealed that male nurses were more likely to experience higher levels of technophobia compared to their female counterparts. This result is different from some previous studies, such as a study of 176 South African university students, which found no significant association between gender and technophobia [34]. Kotzé et al. also reported that, women historically exhibited higher levels of anxiety toward technology adoption, but the gender gap has narrowed in recent years [35]. However, there are also studies that have found that women were more positive than men about the impact of technology on people and their work environments, and women also reflected greater comfort in using computers than men [36]. These discrepancies may be explained by the differing contexts and populations studied. It is possible that specific professional settings, such as nursing, introduce unique stressors that exacerbate technophobia for male nurses. In many nursing work environments, when technical issues arise—such as system updates, troubleshooting, or the implementation of new technologies—male nurses may be more likely to be expected to assist with managing these issues due to societal expectations or perceived expertise in handling technology [37, 38]. Although this possibility was not directly measured in the present study, such expectations might contribute to additional role‐related pressure when new systems or technologies are introduced quickly, without sufficient time for familiarization [39]. Therefore, nursing managers should consider implementing a more balanced distribution of technical tasks and provide targeted support and training for all nursing staff, regardless of gender, to foster a more inclusive and effective work environment.

Family responsibilities, particularly parenthood, may also be associated with technophobia [40]. In this study, nurses with one child showed more intention to experience significantly higher levels of technophobia compared to their childless counterparts. This is in agreement with the findings of Agota, who uncovered that teleworkers with children experience more negative impacts on their work performance and emotional well‐being compared to those without children [40]. Nursing is often characterized by shift work and high‐intensity demands, which limit the time available for professional development [41]. This is particularly true for nurses with children, as their time for learning and adapting to new technologies is further restricted by family responsibilities, exacerbating feelings of inadequacy when confronted with new technologies. Additionally, the dual pressures of parenting and professional duties can deplete cognitive and emotional resources [42]. Nurses with children may experience increased stress from balancing childcare with the high demands of their work, including mastering new technologies. This emotional burden can diminish their motivation to engage with new technological tools. This finding underscores the need for nursing managers to consider the unique challenges faced by nurses with children when implementing technology adoption strategies. Providing flexible training schedules, childcare support, and targeted resources for these nurses may help alleviate technophobia and promote more effective engagement with new technologies, ultimately improving their professional development.

The study also found that living alone is associated with higher levels of technophobia, while higher levels of social support are linked to lower levels of technophobia among nurses. However, the observed effect sizes were relatively small, especially for social support, suggesting that these associations may have limited practical implications. This finding is consistent with previous studies that not living alone and social support represent important means of alleviating technophobia among Chinese older adults [15]. In nurse populations, the role of social support in alleviating work‐related stress and enhancing work engagement has been well established [43, 44], and these are vital prerequisites to learn new technologies. Nursing requires a high level of concentration and precision, and this constant vigilance demands not only technical skills but also significant psychological and emotional focus. Due to the lack of immediate social support, nurses who live alone may experience greater feelings of isolation, which can exacerbate the stress and anxiety related to the adoption of new technologies, particularly in high‐pressure environments like healthcare [45]. This finding also echoes the stress‐buffering model, which posits that social support can reduce or eliminate the stress reaction [46]. Furthermore, living alone may limit nurses’ opportunities for collaborative learning, which is often a key aspect of professional development in healthcare settings. Nurses with strong social support networks, whether from family, friends, or colleagues, are more likely to benefit from emotional encouragement [47]. They are also more likely to have opportunities to share knowledge, discuss challenges, and build confidence in using new technologies through informal social networks. These interactions can enhance nurses’ sense of security and competence when engaging with new technological tools, thereby improving their ability to cope with the demands of new technologies.

The study found that heavier workloads are associated with higher levels of technophobia, while higher job satisfaction is linked to lower levels of technophobia among nurses. These findings underscore the significant influence of work‐related factors on nurses’ attitudes toward technology. A recent study indicated that in major general hospitals in China, the nurse‐to‐patient ratio averages 1:8.0 during daytime hours, with each nurse responsible for the care of eight patients, and this ratio increases to 1:23 at night [48]. Nurses with heavy workloads often experience increased stress and cognitive overload, leaving them with fewer resources—both emotional and cognitive—to engage with new technologies, as new technologies are perceived as an additional burden in an already demanding work environment [49]. In contrast, job satisfaction is defined as “the pleasurable emotional state resulting from the appraisal of one’s job as achieving or facilitating the achievement of one’s job values [50]”. Previous studies have shown that job satisfaction mediated the relationship between occupational stressors and burnout among Chinese nurses [51]. Higher job satisfaction often reflects a supportive work environment, strong social networks, and a sense of accomplishment, all of which can reduce technophobia by enhancing self‐efficacy and fostering a positive attitude toward professional growth [52]. Nurses who are satisfied with their work environment are more likely to view technological challenges as opportunities for growth, rather than sources of stress; thus, these nurses may have more energy and motivation to engage with new technologies.

Insomnia status was an independent factor associated with technophobia. Especially for nurses working long‐term night shifts or rotating shifts, irregular work hours may disrupt sleep patterns and contribute to poor sleep quality [53]. Poor sleep quality and sleep deprivation may impair cognitive performance, including attention, memory, and emotional regulation, which are important for learning and adapting to new technologies [54]. Previous studies have shown that cognitive function in nurses significantly declines during consecutive night shifts, reaching levels similar to those of being drunk (> 0.08 blood alcohol content) by the end of a 12‐h shift [18]. These cognitive challenges may increase the perceived difficulty of using unfamiliar technological systems and thereby contribute to technophobia. Therefore, nursing managers should implement strategies such as organizing shift schedules and ensuring adequate rest and recovery time to help improve nurses’ insomnia [55], thereby enhancing their ability to learn and adapt to new technologies and reducing technophobia.

There are also some limitations in the current study. First, the cross‐sectional design of this study makes it difficult to establish cause‐and‐effect relationships. Second, although the study includes multiple centers, they are all concentrated in authoritative third‐class hospitals within the province, and the findings may not be generalizable to all healthcare institutions, particularly in rural or less technologically advanced areas. Third, data were collected through online questionnaires distributed via WeChat, which may introduce potential self‐report bias, such as social desirability bias or recall bias. Fourth, the Technophobia Scale was originally validated among older adults rather than nurses. However, it has subsequently been applied in several adult populations and demonstrated excellent internal consistency in the present study (Cronbach’s *α* = 0.984). Moreover, the scale items capture general negative emotional reactions to technology and are not specifically tailored to older adults, suggesting their applicability to broader adult populations. Fifth, the sample consisted predominantly of female nurses (96.99%), which may limit the robustness of gender comparisons. Future studies may consider recruiting a more balanced sample to better explore potential gender differences. Sixth, this study did not assess nurses’ digital literacy or technological competence, which may also influence technophobia and should be considered in future research. Finally, although several factors were statistically associated with technophobia, some associations showed relatively small effect sizes (e.g., social support), which may limit their practical significance.

## 5. Conclusion

This multicenter cross‐sectional study demonstrated that technophobia among nurses in China was at a low‐to‐moderate level. Male nurses and those who have a child or live alone, as well as nurses reporting heavier workloads, poorer sleep quality, lower job satisfaction, and weaker social support, tended to exhibit greater technology‐related fear. These findings highlight the need for nursing managers to create supportive work environments and implement practical interventions such as structured digital literacy training programs, flexible scheduling arrangements for nurses with childcare responsibilities, and peer mentoring initiatives to strengthen nurses’ digital competence, facilitate their adaptation to technological change, and ultimately enhance the quality of patient care.

## Author Contributions

Xuange Sun: methodology, conceptualization, validation, formal analysis, data curation, visualization, writing–original draft, writing–review and editing, and software.

Xu Liu: conceptualization, validation, formal analysis, data curation, visualization, writing–original draft, writing–review and editing, and software.

Xiaolin Chang: validation, investigation, resources, and writing–review and editing.

Na Hu: writing–review and editing.

Xiangxiu Qi: investigation, resources, writing–review and editing, and supervision.

Xiaofei Li: investigation, resources, writing–review and editing, and supervision.

## Funding

No funding was received for this manuscript.

## Disclosure

The authors reviewed and assume full responsibility for the manuscript’s final version.

## Ethics Statement

Ethical approval for this study was obtained from the Ethics Committee of the First Hospital of China Medical University, number [2025] 782. The study was conducted in accordance with the Declaration of Helsinki. Informed consent was obtained from all individual participants included in the study.

## Consent

Please see the Ethics Statement.

## Conflicts of Interest

The authors declare no conflicts of interest.

## Data Availability

The data that support the findings of this study are available from the corresponding authors upon reasonable request.
